# Supplementary material for: Hybrid Models and Biological Model Reduction with PyDSTool
Source: PLoS Comput Biol. 2012 Aug 9;8(8):e1002628. doi: 10.1371/journal.pcbi.1002628 (PMC3415397; doi:10.1371/journal.pcbi.1002628)
Supplement: Text S4 — Complete source code for the PyDSTool package (version 0.88.120504). Includes API documentation and help files linking to web pages. This file is identical to the current public release on Sourceforge.net. (ZIP) [file pcbi.1002628.s004.zip › PyDSTool/html/PyDSTool.Model.domain_test-class.html]

xml version="1.0" encoding="ascii"?


PyDSTool.Model.domain\_test


| Home | Trees | Indices | Help | PyDSTool |
| --- | --- | --- | --- | --- |

Package PyDSTool ::
Module Model ::
Class domain\_test

[hide private]

[frames] | no frames]

# Class domain\_test

source code

```
       object --+            
                |            
 MProject.feature --+        
                    |        
MProject.feature_node --+    
                        |    
 MProject.qt_feature_node --+
                            |
                           domain_test
```

---


|  |  |
| --- | --- |
| Instance Methods | [hide private] |

|  |  |
| --- | --- |
| \_\_init\_\_(self, name, description=`'``'`, pars=None)  Sub-features is an ordered sequence of QL or QT feature instances which are (by default) evaluated in this order on a trajectory segment unless evaluation method is overridden. | source code |

|  |  |
| --- | --- |
| evaluate(self, traj)  Default method: evaluate sub-features in order (assumes they are stored as a list). | source code |

|  |  |
| --- | --- |
| \_find\_idx(self)  Helper function for finding lowest index in trajectory meshpoints at which domain test first failed | source code |

**Inherited from `MProject.feature_node`**:
`__getitem__`,
`__repr__`,
`__str__`,
`propagate_verbosity`,
`set_ref_traj`

**Inherited from `MProject.feature_node`** (private):
`_residual_info`

**Inherited from `MProject.feature`**:
`__call__`,
`__eq__`,
`__hash__`,
`__ne__`,
`finish`,
`info`,
`postprocess_ref_traj`,
`prepare`,
`reset_metric`,
`validate`

**Inherited from `object`**:
`__delattr__`,
`__getattribute__`,
`__new__`,
`__reduce__`,
`__reduce_ex__`,
`__setattr__`


|  |  |
| --- | --- |
| Properties | [hide private] |

**Inherited from `object`**:
`__class__`


|  |  |
| --- | --- |
| Method Details | [hide private] |

|  |  |
| --- | --- |
| \_\_init\_\_(self, name, description=`'``'`, pars=None)  *(Constructor)* | source code |

Sub-features is an ordered sequence of QL or QT feature instances
which are (by default) evaluated in this order on a trajectory segment
unless evaluation method is overridden.

For more sophisticated use of sub-features, they should be provided as
a dictionary mapping feature names to the feature instance.

Overrides: object.\_\_init\_\_: *(inherited documentation)*

|  |  |
| --- | --- |
| evaluate(self, traj) | source code |

Default method: evaluate sub-features in order (assumes they are
stored as a list).

Can override with more sophisticated method (e.g. for use with a
dictionary of sub-features).

Overrides: MProject.feature.evaluate

|  |  |
| --- | --- |
| \_find\_idx(self) | source code |

Helper function for finding lowest index in trajectory meshpoints at
which domain test first failed

Overrides: MProject.feature.\_find\_idx

  


| Home | Trees | Indices | Help | PyDSTool |
| --- | --- | --- | --- | --- |

|  |  |
| --- | --- |
| Generated by Epydoc 3.0.1 on Fri May 4 15:24:07 2012 | http://epydoc.sourceforge.net |
